# Crystal structure and hydrogen bonding in the water-stabilized proton-transfer salt brucinium 4-amino­phenyl­arsonate tetra­hydrate

**DOI:** 10.1107/S2056989016006691

**Published:** 2016-04-29

**Authors:** Graham Smith, Urs D. Wermuth

**Affiliations:** a Science and Engineering Faculty, Queensland University of Technology, GPO Box 2434, Brisbane, Queensland 4001, Australia

**Keywords:** crystal structure, brucinium salts, *p*-arsanilic acid, water stabilization, hydrogen bonding

## Abstract

In the structure of the brucinium salt of 4-amino­phenyl­arsonic acid (*p*-arsanilic acid), systematically 2,3-dimeth­oxy-10-oxostrychnidinium 4-amino­phenyl­ar­son­ate tetra­hydrate, (C_23_H_27_N_2_O_4_)[As(C_6_H_7_N)O_2_(OH)]·4H_2_O, the brucinium cations form the characteristic undulating and overlapping head-to-tail layered brucine substructures packed along [010]. The arsanilate anions and the water mol­ecules of solvation are accommodated between the layers and are linked to them through a primary cation N—H⋯O(anion) hydrogen bond, as well as through water O—H⋯O hydrogen bonds to brucinium and arsanilate ions as well as bridging water O-atom acceptors, giving an overall three-dimensional network structure.

## Abstract 2

The tetra­hydrated brucinium salt of *p*-arsanilic acid forms a three-dimensional hydrogen-bonded network featuring the previously described undulating layered brucinium host substructure accommodating the anions and water guest mol­ecules and stabilized by cation–anion N—H⋯O and O—H⋯O hydrogen-bonding inter­actions.

## Chemical context   

The *Strychnos* alkaloid base brucine, (2,3-di­meth­oxy­strychnidin-10-one; BRU) has been extensively employed as a resolving agent for chiral organic compounds (Wilen, 1972 ▸). With chiral acids, the separation is achieved through proton-transfer to N19 of the strychnidine cage (p*K*_a2_ = 11.7; O’Neil, 2001 ▸), followed by separation of the resultant crystalline salt products by fractional crystallization. Similar effects are achieved with the essentially identical *Strychnos* alkaloid strychnine but separation efficiency favours brucine. This is probably because of the formation in the crystal of characteristic brucinium host substructures comprising head-to-tail undulating layers of brucine mol­ecules or cations which accommodate selectively the hydrogen-bonded guest mol­ecules in the crystal structure. A characteristic of the substructure is the repeat inter­val in the layer of *ca* 12.3 Å along a 2_1_ screw axis in the crystal, which is reflected in the unit-cell dimension, with brucine being predominantly in the monoclinic space group *P*2_1_ or the ortho­rhom­bic space group *P*2_1_2_1_2_1_ (Smith, Wermuth & White, 2006 ▸; Smith, Wermuth, Young & White, 2006 ▸).
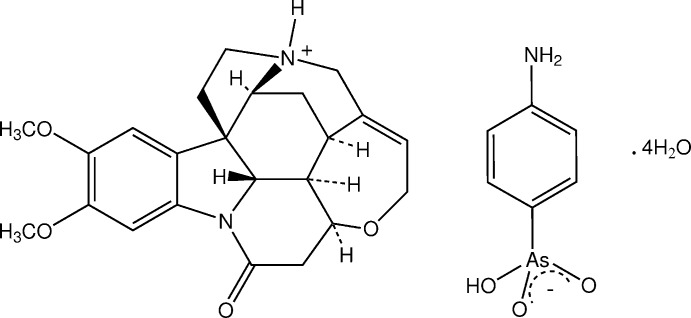


This example of mol­ecular recognition was described in the early structure determinations of brucinium benzoyl-d-alanin­ate (Gould & Walkinshaw, 1984 ▸) and in the structures of the pseudopolymorphic brucine solvates, brucine–MeOH (1:1) and brucine–EtOH–water (1/1/2) (Glover *et al.*, 1985 ▸). The guest mol­ecules are accommodated inter­stitially within the layers and are commonly accompanied by compatible polar solvent mol­ecules, usually generating high-dimensional hydrogen-bonded crystal structures.

Currently, a large number of structures of brucine compounds with chiral organic mol­ecules, including both acids and non-acids are known, but in addition those with achiral compounds also feature. Of inter­est to us have been the structures of brucinium proton-transfer salts with largely simple organic acids, prepared under aqueous alcoholic conditions, the crystalline products being stabilized by solvent mol­ecules. Water-stabilized achiral carboxyl­ate examples include BRU^+^ hydrogen fumarate^−^·1.5H_2_O (Dijksma, Gould, Parsons & Walkinshaw, 1998 ▸), BRU^+^ di­hydrogen citrate^−^·3H_2_O (Smith, Wermuth & White, 2005 ▸) and BRU^+^ benzo­ate^−^·3H_2_O (Białońska & Ciunik, 2006*b* ▸).

Other organic acids besides carboxyl­ates may be included among the set but fewer structural examples are known, *e.g*. sulfonates (BRU^+^ toluene-4-sulfonate^−^·3H_2_O; Smith, Wermuth, Healy *et al.*, 2005 ▸). However, no brucinium arsonate structures are known, so that the reaction of brucine with 4-amino­phenyl­arsonic acid (*p*-arsanilic acid) in 2-propanol/water was carried out, resulting in the formation of the crystalline hydrated title salt, C_23_H_27_N_2_O_4_^+^· C_6_H_7_AsNO_3_^−^·4H_2_O, and the structure is reported herein. The acid has biological significance as an anti-helminth in veterinary applications (Thomas, 1905 ▸; Steverding, 2010 ▸) and as a monohydrated sodium salt (atox­yl) which had early usage as an anti-syphilitic (Ehrlich & Bertheim, 1907 ▸; Bosch & Rosich, 2008 ▸). Simple *p*-arsanilate salt structures are not common in the Cambridge Structural Database (Groom *et al.*, 2016 ▸), with only the NH_4_^+^ and K^+^ salts (Smith & Wermuth, 2014 ▸) and the guanidinium salts (Smith & Wermuth, 2010 ▸; Latham *et al.*, 2011 ▸) being known.

## Structural commentary   

**Figure 1:**
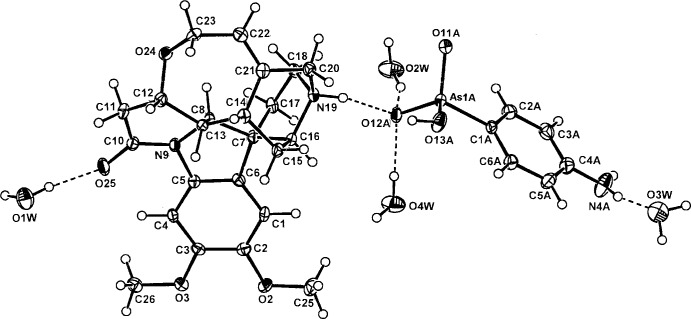
Mol­ecular configuration and atom-numbering scheme for the brucinium cation, *p*-arsanilate anion *A* and the four water mol­ecules of solvation in the asymmetric unit of the title salt. Inter-species hydrogen bonds are shown as dashed lines. Non-H atoms are shown as 40% probability displacement ellipsoids.

The asymmetric unit of the title salt comprises a brucinium cation, a *p*-arsanilate anion *A* and four water mol­ecules of solvation, (O1*W*–O4*W*), all inter-associated through hydrogen bonds (Fig. 1 ▸). Protonation has occurred as expected at N19 of the brucine cage, the invoked Peerdeman (1956 ▸) absolute configuration for the strychnidinium mol­ecule giving the overall Cahn–Ingold stereochemistry of the cation as C7(*R*), C8(*S*), C12(*S*), C13(*R*), C14(*R*), C16(*S*) and the additional introduced (*S*) chiral centre at N19.

## Supra­molecular features   

**Figure 2:**
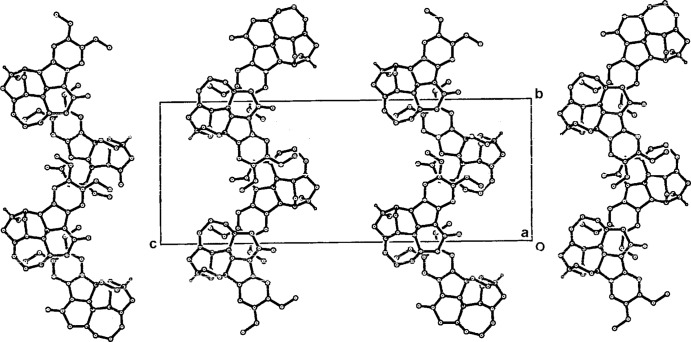
The undulating brucinium sheet substructures in the unit cell of the title salt, less the inter-sheet anion and water mol­ecules, viewed down *a*. All H atoms except that of the protonated N19 atom have also been removed.

**Figure 3:**
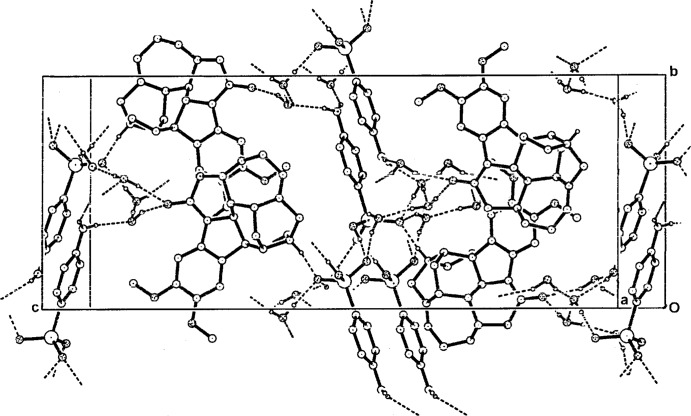
A perspective view of the packing in the unit cell, viewed along the approximate *a*-axial direction, showing the associated anions and the water mol­ecules in the inter­stitial regions of the brucinium layered substructures, with hydrogen-bonding inter­actions shown as dashed lines.

The brucinium cations form into the previously described undulating sheet–host substructures which are considered to be the reason for the mol­ecular recognition peculiar to brucine (Gould & Walkinshaw, 1984 ▸; Gould *et al.*, 1985 ▸; Dijksma, Gould, Parsons & Walkinshaw, 1998 ▸; Dijksma, Gould, Parsons, Taylor & Walkinshaw, 1998 ▸; Oshikawa *et al.*, 2002 ▸; Białońska & Ciunik, 2004 ▸). In the title salt, these substructures extend along the *b-*axis direction, with the previously described 2_1_ propagation of the brucinium cations along the *ca* 12.3 Å axis (Fig. 2 ▸). The *p*-arsanilate anions and the water mol­ecules occupy the inter­stitial spaces in the structure. The protonated N19 atom of the cation gives a single hydrogen-bonding inter­action with a *p*-arsanilate oxygen acceptor (O12*A*) while two of the solvent water mol­ecules (O1*W* and O3*W*) form hydrogen bonds with the carbonyl O25 atom of the the brucinium cation (Table 1 ▸). Within the inter-sheet channels, the *p*-arsanilate anions are linked head-to-head through an O13*A*—H⋯O11*A* ^ii^ hydrogen bond while both H atoms of the amine group form hydrogen bonds with water mol­ecules O3*W* and O4*W* ^i^. The water mol­ecules O2*W* and O4*A* are further linked to the *p*-arsanilate O-atom O12*A* with O2*W* also linked to O11*A* ^iv^. Water mol­ecules O3*W* and O4*W* ^i^ give inter-water hydrogen bonds and together with a number of inter-mol­ecular C—H⋯O inter­actions (Table 1 ▸) result in an overall three-dimensional network structure (Fig. 3 ▸).

## Database survey   

Inter­stitial water mol­ecules are present in the structures of the brucine pseudo-polymorphic structures, *e.g*. the common tetra­hydrate form and the 5.2 hydrate (Smith *et al.*, 2006*a* ▸) and the dihydrate (Smith *et al.*, 2007 ▸), as well as the mixed solvates BRU–EtOH–H_2_O (1/1/2) (Glover *et al.*, 1985 ▸) and BRU–*i*-PrOH–H_2_O (1/1/2) (Białońska & Ciunik, 2004 ▸). A large number of water-stabilized brucinium salts of acids are known: with the inorganic sulfate (BRU)_2_SO_4_·7H_2_O (Białońska & Ciunik, 2005 ▸) and most commonly with aromatic carboxyl­ates, *e.g*. the benzoate (a trihydrate; Białońska & Ciunik, 2006*b* ▸); the 4-nitro­benzoate (a dihydrate; Białońska & Ciunik, 2007 ▸); the 3,5-di­nitro­benzoate (a trihydrate; Białońska & Ciunik, 2006*a* ▸); the 3,5-di­nitro­salicylate (a monohydrate; Smith *et al.*, 2006*a* ▸); the phthalate (a monohydrate; Krishnan, Gayathri, Sivakumar, Gunasekaran & Anbalagen, 2013 ▸); the hydrogen isophthalate (a trihydrate; Smith, Wermuth, Young & White, 2006 ▸); the hydrogen 3-nitro­phthalate (a dihydrate; Smith, Wermuth, Young & Healy, 2005 ▸) and the picramino­benzoate (a monohydrate; Smith & Wermuth, 2011 ▸).

Aliphatic carboxyl­ate examples are: with hydrogen oxalate (a dihydrate; Krishnan, Gayathri, Sivakumar, Chakkaravathi & Anbalagen, 2013 ▸); with hydrogen fumarate (a sesquihydrate; Dijksma, Gould, Parsons & Walkinshaw, 1998 ▸); with hydrogen (*S*)-malate (a penta­hydrate; Smith, Wermuth & White, 2006 ▸); with di­hydrogen citrate (a trihydrate; Smith, Wermuth & White, 2005 ▸); with l-glycerate (a 4.75 hydrate; Białońska *et al.*, 2005 ▸) and with hydrogen *cis*-cyclo­hexane-1,2-di­carboxyl­ate (a dihydrate; Smith *et al.*, 2012 ▸). Some sulfonate salts are also known, *e.g*. with toluene-4-sulfonate (a trihydrate; Smith, Wermuth, Healy *et al.*, 2005 ▸); with 3-carb­oxy-4-hy­droxy­benzene­sulfonate (a penta­hydrate; Smith *et al.*, 2006*b* ▸) and with biphenyl-4,4′-di­sulfonate (a hexa­hydrate; Smith *et al.*, 2010 ▸).

## Synthesis and crystallization   

The title compound was synthesized by heating together under reflux for 10 min, 1 mmol qu­anti­ties of brucine tetra­hydrate and 4-amino­phenyl­arsonic acid in 50 mL of 80% 2-propanol/water. After concentration to *ca* 30 mL, partial room-temperature evaporation of the hot-filtered solution gave thin colourless crystal plates of the title compound from which a specimen was cleaved for the X-ray analysis.

## Refinement details   

Crystal data, data collection and structure refinement details are summarized in Table 2 ▸. Hydrogen atoms potentially involved in hydrogen-bonding inter­actions were located by difference methods but their positional parameters were constrained in the refinement with N—H and O—H = 0.90 Å, and with *U*_iso_(H) = 1.2*U*_eq_(N) or 1.5*U*_eq_(O). Other H atoms were included in the refinement at calculated positions [C—H(aromatic) = 0.95 Å and C—H (aliphatic) = 0.97–1.00 Å] and treated as riding with *U*_iso_(H) = 1.2*U*_eq_(C). The absolute configuration determined for the parent strychnidinin-10-one mol­ecule (Peerdeman, 1956 ▸) was invoked and was confirmed in the the structure refinement.

## Supplementary Material

Crystal structure: contains datablock(s) global, I. DOI: 10.1107/S2056989016006691/lh5811sup1.cif

Structure factors: contains datablock(s) I. DOI: 10.1107/S2056989016006691/lh5811Isup2.hkl

CCDC reference: 1475231

Additional supporting information:  crystallographic information; 3D view; checkCIF report

## Figures and Tables

**Table 1:** Hydrogen-bond geometry (Å, °)

*D*—H⋯*A*	*D*—H	H⋯*A*	*D*⋯*A*	*D*—H⋯*A*
N19—H19⋯O12*A*	0.91 (4)	1.72 (4)	2.610 (3)	168 (4)
N4*A*—H41*A*⋯O4*W* ^i^	0.89 (3)	2.46 (4)	3.291 (5)	155 (4)
N4*A*—H42*A*⋯O3*W*	0.90 (3)	2.25 (3)	3.137 (6)	169 (4)
O13*A*—H13*A*⋯O11*A* ^ii^	0.90 (4)	1.67 (4)	2.546 (3)	165 (4)
O1*W*—H11*W*⋯O25	0.90 (4)	1.95 (4)	2.843 (4)	175 (3)
O1*W*—H12*W*⋯O2*W* ^iii^	0.90 (3)	1.87 (4)	2.760 (5)	168 (4)
O2*W*—H21*W*⋯O12*A*	0.90 (3)	2.11 (3)	2.945 (4)	153 (4)
O2*W*—H22*W*⋯O11*A* ^iv^	0.89 (3)	2.07 (4)	2.915 (4)	158 (5)
O3*W*—H31*W*⋯O25^v^	0.91 (4)	2.06 (4)	2.922 (4)	159 (3)
O3*W*—H32*W*⋯O4*W* ^vi^	0.91 (3)	1.91 (3)	2.791 (4)	164 (3)
O4*W*—H41*W*⋯O1*W* ^vii^	0.90 (4)	1.88 (4)	2.770 (5)	172 (5)
O4*W*—H42*W*⋯O12*A*	0.89 (4)	1.91 (4)	2.802 (4)	174 (5)
C14—H14⋯O3^viii^	1.00	2.52	3.363 (4)	142
C15—H151⋯O11*A* ^ii^	0.99	2.60	3.561 (4)	165
C18—H182⋯O2*W*	0.99	2.58	3.422 (5)	143
C20—H201⋯O11*A* ^ii^	0.99	2.41	3.388 (4)	170
C20—H202⋯O13*A* ^iv^	0.99	2.43	3.229 (4)	137

Symmetry codes: (i) 
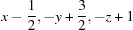
; (ii) 
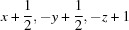
; (iii) 
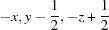
; (iv) 
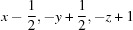
; (v) 
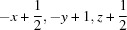
; (vi) 
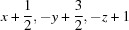
; (vii) 
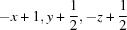
; (viii) 
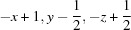
.

**Table 2:** Experimental details

Crystal data	
Chemical formula	(C_23_H_27_N_2_O_4_)[As(C_6_H_7_N)O_2_(OH)]·4H_2_O
*M*_r_	683.58
Crystal system, space group	Orthorhombic, *P*2_1_2_1_2_1_
Temperature (K)	200
*a*, *b*, *c* (Å)	7.6553 (3), 12.3238 (5), 31.960 (2)
*V* (Å^3^)	3015.2 (3)
*Z*	4
Radiation type	Mo *K*α
μ (mm^−1^)	1.19
Crystal size (mm)	0.36 × 0.34 × 0.10

Data collection	
Diffractometer	Oxford Diffraction Gemini-S CCD-detector diffractometer
Absorption correction	Multi-scan (*CrysAlis PRO*; Rigaku OD, 2015 ▸)
*T*_min_, *T*_max_	0.811, 0.980
No. of measured, independent and observed [*I* > 2σ(*I*)] reflections	11983, 6980, 5901
*R*_int_	0.032
(sin θ/λ)_max_ (Å^−1^)	0.693

Refinement	
*R*[*F* ^2^ > 2σ(*F* ^2^)], *wR*(*F* ^2^), *S*	0.048, 0.096, 1.05
No. of reflections	6980
No. of parameters	433
No. of restraints	14
H-atom treatment	H atoms treated by a mixture of independent and constrained refinement
Δρ_max_, Δρ_min_ (e Å^−3^)	0.55, −0.46
Absolute structure	Flack (1983 ▸), 3672 Friedel pairs
Absolute structure parameter	−0.005 (9)

Computer programs: *CrysAlis PRO* (Rigaku OD, 2015 ▸), *SIR92* (Altomare *et al.*, 1993 ▸), *SHELXL97* (Sheldrick, 2008 ▸) within *WinGX* (Farrugia, 2012 ▸) and *PLATON* (Spek, 2009 ▸).

## supplementary crystallographic information

### Crystal data

**Table:**

(C_23_H_27_N_2_O_4_)[As(C_6_H_7_N)O_2_(OH)]·4H_2_O	*F*(000) = 1432
*M**_r_* = 683.58	*D*_x_ = 1.506 Mg m^−^^3^
Orthorhombic, *P*2_1_2_1_2_1_	Mo *K*α radiation, λ = 0.71073 Å
Hall symbol: P 2ac 2ab	Cell parameters from 2822 reflections
*a* = 7.6553 (3) Å	θ = 3.4–27.9°
*b* = 12.3238 (5) Å	µ = 1.19 mm^−^^1^
*c* = 31.960 (2) Å	*T* = 200 K
*V* = 3015.2 (3) Å^3^	Plate, colourless
*Z* = 4	0.36 × 0.34 × 0.10 mm

### Data collection

**Table:**

Oxford Diffraction Gemini-S CCD-detector diffractometer	6980 independent reflections
Radiation source: Enhance (Mo) X-ray source	5901 reflections with *I* > 2σ(*I*)
Graphite monochromator	*R*_int_ = 0.032
Detector resolution: 16.077 pixels mm^-1^	θ_max_ = 29.5°, θ_min_ = 3.1°
ω scans	*h* = −10→6
Absorption correction: multi-scan (*CrysAlis PRO*; Rigaku OD, 2015)	*k* = −16→16
*T*_min_ = 0.811, *T*_max_ = 0.980	*l* = −43→25
11983 measured reflections	

### Refinement

**Table:**

Refinement on *F*^2^	Secondary atom site location: difference Fourier map
Least-squares matrix: full	Hydrogen site location: inferred from neighbouring sites
*R*[*F*^2^ > 2σ(*F*^2^)] = 0.048	H atoms treated by a mixture of independent and constrained refinement
*wR*(*F*^2^) = 0.096	*w* = 1/[σ^2^(*F*_o_^2^) + (0.0414*P*)^2^ + 0.2011*P*] where *P* = (*F*_o_^2^ + 2*F*_c_^2^)/3
*S* = 1.05	(Δ/σ)_max_ = 0.001
6980 reflections	Δρ_max_ = 0.55 e Å^−^^3^
433 parameters	Δρ_min_ = −0.46 e Å^−^^3^
14 restraints	Absolute structure: Flack (1983), 3672 Friedel pairs
Primary atom site location: structure-invariant direct methods	Absolute structure parameter: −0.005 (9)

### Special details

**Table:**

Geometry. Bond distances, angles etc. have been calculated using the rounded fractional coordinates. All su's are estimated from the variances of the (full) variance-covariance matrix. The cell esds are taken into account in the estimation of distances, angles and torsion angles
Refinement. Refinement of F^2^ against ALL reflections. The weighted R-factor wR and goodness of fit S are based on F^2^, conventional R-factors R are based on F, with F set to zero for negative F^2^. The threshold expression of F^2^ > 2sigma(F^2^) is used only for calculating R-factors(gt) etc. and is not relevant to the choice of reflections for refinement. R-factors based on F^2^ are statistically about twice as large as those based on F, and R- factors based on ALL data will be even larger.

### Fractional atomic coordinates and isotropic or equivalent isotropic displacement parameters (Å^2^)

**Table:**

	*x*	*y*	*z*	*U*_iso_*/*U*_eq_	
O2	0.2664 (3)	0.56076 (19)	0.24172 (7)	0.0288 (8)	
O3	0.2324 (3)	0.44912 (19)	0.17363 (7)	0.0251 (7)	
O24	0.2010 (3)	−0.13571 (17)	0.32202 (7)	0.0224 (7)	
O25	0.2193 (4)	0.0496 (2)	0.19539 (7)	0.0336 (9)	
N9	0.1591 (3)	0.1193 (2)	0.25929 (8)	0.0192 (7)	
N19	0.1326 (4)	0.2044 (2)	0.39973 (8)	0.0220 (8)	
C1	0.2107 (4)	0.4025 (3)	0.28549 (10)	0.0220 (10)	
C2	0.2316 (4)	0.4525 (3)	0.24709 (10)	0.0206 (10)	
C3	0.2176 (4)	0.3914 (3)	0.21006 (10)	0.0200 (9)	
C4	0.1912 (4)	0.2806 (2)	0.21125 (9)	0.0192 (9)	
C5	0.1762 (5)	0.2319 (2)	0.25023 (10)	0.0186 (9)	
C6	0.1822 (5)	0.2909 (3)	0.28690 (9)	0.0200 (9)	
C7	0.1402 (4)	0.2196 (3)	0.32382 (10)	0.0194 (9)	
C8	0.1639 (4)	0.1035 (3)	0.30544 (9)	0.0178 (9)	
C10	0.2084 (5)	0.0380 (3)	0.23349 (10)	0.0224 (10)	
C11	0.2482 (5)	−0.0701 (3)	0.25362 (11)	0.0244 (11)	
C12	0.3195 (5)	−0.0703 (3)	0.29876 (10)	0.0216 (10)	
C13	0.3369 (4)	0.0468 (3)	0.31477 (9)	0.0173 (9)	
C14	0.3946 (4)	0.0634 (3)	0.36027 (10)	0.0208 (10)	
C15	0.4243 (4)	0.1858 (3)	0.36540 (11)	0.0217 (10)	
C16	0.2486 (5)	0.2415 (3)	0.36351 (10)	0.0215 (10)	
C17	−0.0479 (4)	0.2361 (3)	0.33974 (11)	0.0236 (11)	
C18	−0.0461 (4)	0.1812 (3)	0.38190 (10)	0.0236 (10)	
C20	0.2066 (5)	0.1088 (3)	0.42293 (9)	0.0234 (10)	
C21	0.2646 (4)	0.0242 (3)	0.39246 (10)	0.0221 (10)	
C22	0.2076 (5)	−0.0761 (3)	0.39424 (10)	0.0235 (10)	
C23	0.2581 (5)	−0.1618 (3)	0.36323 (11)	0.0269 (11)	
C25	0.2845 (6)	0.6248 (3)	0.27850 (12)	0.0400 (14)	
C26	0.2222 (4)	0.3880 (3)	0.13581 (10)	0.0263 (10)	
As1A	0.18853 (4)	0.38087 (2)	0.50015 (1)	0.0194 (1)	
O11A	0.0706 (3)	0.2967 (2)	0.52906 (7)	0.0288 (8)	
O12A	0.1351 (3)	0.37219 (19)	0.44956 (7)	0.0256 (7)	
O13A	0.4046 (3)	0.3544 (2)	0.50798 (9)	0.0361 (9)	
N4A	0.1284 (6)	0.8469 (3)	0.55939 (14)	0.0526 (15)	
C1A	0.1723 (5)	0.5265 (2)	0.51885 (9)	0.0213 (9)	
C2A	0.0081 (5)	0.5733 (3)	0.52485 (11)	0.0277 (11)	
C3A	−0.0043 (6)	0.6792 (3)	0.53827 (11)	0.0320 (12)	
C4A	0.1423 (6)	0.7411 (3)	0.54628 (12)	0.0314 (13)	
C5A	0.3047 (6)	0.6939 (3)	0.53962 (11)	0.0324 (11)	
C6A	0.3193 (5)	0.5885 (3)	0.52554 (10)	0.0271 (10)	
O1W	0.4311 (4)	−0.0600 (3)	0.13578 (10)	0.0461 (11)	
O2W	−0.2441 (4)	0.3881 (3)	0.43528 (11)	0.0521 (11)	
O3W	0.4514 (4)	0.8770 (3)	0.61869 (11)	0.0587 (12)	
O4W	0.2795 (4)	0.5374 (3)	0.40023 (10)	0.0511 (11)	
H1	0.21570	0.44380	0.31060	0.0260*	
H4	0.18370	0.23920	0.18630	0.0230*	
H8	0.06440	0.05630	0.31430	0.0210*	
H12	0.43720	−0.10550	0.29900	0.0260*	
H13	0.42710	0.08270	0.29690	0.0210*	
H14	0.50800	0.02480	0.36480	0.0250*	
H16	0.26740	0.32150	0.36610	0.0260*	
H19	0.122 (6)	0.258 (3)	0.4190 (11)	0.0620*	
H22	0.13050	−0.09540	0.41630	0.0280*	
H111	0.33410	−0.10810	0.23570	0.0290*	
H112	0.13960	−0.11370	0.25330	0.0290*	
H151	0.48150	0.20080	0.39260	0.0260*	
H152	0.50090	0.21300	0.34270	0.0260*	
H171	−0.07610	0.31420	0.34240	0.0280*	
H172	−0.13360	0.20150	0.32080	0.0280*	
H181	−0.06470	0.10210	0.37890	0.0280*	
H182	−0.13850	0.21120	0.40020	0.0280*	
H201	0.30700	0.13230	0.44020	0.0280*	
H202	0.11670	0.07810	0.44180	0.0280*	
H231	0.38680	−0.17010	0.36320	0.0320*	
H232	0.20630	−0.23190	0.37180	0.0320*	
H251	0.30870	0.70020	0.27070	0.0600*	
H252	0.38120	0.59660	0.29540	0.0600*	
H253	0.17600	0.62160	0.29470	0.0600*	
H261	0.23380	0.43690	0.11180	0.0390*	
H262	0.10930	0.35070	0.13440	0.0390*	
H263	0.31660	0.33430	0.13520	0.0390*	
H2A	−0.09470	0.53230	0.51970	0.0330*	
H3A	−0.11650	0.71040	0.54210	0.0390*	
H5A	0.40740	0.73490	0.54490	0.0390*	
H6A	0.43160	0.55850	0.52040	0.0330*	
H13A	0.445 (6)	0.298 (3)	0.4931 (13)	0.0770*	
H41A	0.022 (3)	0.876 (4)	0.5617 (15)	0.0620*	
H42A	0.227 (3)	0.861 (4)	0.5735 (13)	0.0620*	
H11W	0.360 (5)	−0.029 (4)	0.1548 (10)	0.0770*	
H12W	0.358 (5)	−0.071 (4)	0.1141 (10)	0.0770*	
H21W	−0.134 (3)	0.406 (4)	0.4425 (16)	0.0770*	
H22W	−0.273 (7)	0.328 (2)	0.4492 (14)	0.0770*	
H31W	0.406 (6)	0.917 (3)	0.6400 (11)	0.0770*	
H32W	0.548 (4)	0.917 (3)	0.6129 (15)	0.0770*	
H41W	0.378 (4)	0.512 (4)	0.3885 (15)	0.0770*	
H42W	0.242 (7)	0.483 (3)	0.4163 (13)	0.0770*	

### Atomic displacement parameters (Å^2^)

**Table:**

	*U*^11^	*U*^22^	*U*^33^	*U*^12^	*U*^13^	*U*^23^
O2	0.0412 (17)	0.0165 (12)	0.0288 (13)	−0.0036 (11)	0.0025 (12)	−0.0002 (10)
O3	0.0292 (13)	0.0255 (13)	0.0207 (12)	−0.0021 (11)	0.0027 (10)	0.0008 (10)
O24	0.0244 (12)	0.0185 (11)	0.0243 (11)	−0.0011 (11)	−0.0001 (11)	0.0000 (9)
O25	0.0533 (19)	0.0281 (14)	0.0195 (12)	0.0026 (14)	0.0064 (13)	−0.0055 (10)
N9	0.0226 (14)	0.0182 (12)	0.0168 (12)	0.0001 (13)	−0.0003 (11)	−0.0035 (11)
N19	0.0261 (15)	0.0230 (15)	0.0169 (14)	−0.0009 (12)	0.0011 (12)	−0.0052 (12)
C1	0.0251 (19)	0.0207 (17)	0.0203 (16)	0.0005 (14)	−0.0011 (15)	−0.0087 (13)
C2	0.0181 (18)	0.0173 (16)	0.0263 (18)	0.0022 (13)	0.0011 (15)	0.0004 (14)
C3	0.0169 (17)	0.0247 (17)	0.0183 (15)	−0.0004 (15)	0.0021 (13)	0.0018 (14)
C4	0.0200 (16)	0.0229 (16)	0.0148 (14)	−0.0001 (15)	0.0007 (15)	−0.0046 (12)
C5	0.0196 (17)	0.0181 (15)	0.0181 (15)	0.0014 (14)	−0.0008 (15)	−0.0012 (12)
C6	0.0196 (16)	0.0213 (15)	0.0192 (15)	0.0022 (15)	0.0023 (15)	0.0000 (13)
C7	0.0228 (17)	0.0176 (16)	0.0177 (16)	0.0017 (13)	0.0007 (14)	−0.0031 (13)
C8	0.0193 (16)	0.0188 (16)	0.0153 (14)	−0.0002 (14)	0.0004 (13)	−0.0039 (12)
C10	0.0214 (18)	0.0235 (17)	0.0224 (17)	−0.0025 (16)	0.0005 (16)	−0.0059 (14)
C11	0.030 (2)	0.0184 (17)	0.0248 (18)	0.0021 (14)	−0.0014 (16)	−0.0066 (14)
C12	0.0204 (17)	0.0190 (16)	0.0255 (17)	0.0035 (16)	0.0024 (16)	−0.0047 (13)
C13	0.0137 (16)	0.0175 (15)	0.0208 (16)	−0.0005 (13)	0.0031 (13)	−0.0038 (12)
C14	0.0164 (17)	0.0248 (18)	0.0211 (17)	0.0020 (14)	−0.0029 (14)	−0.0028 (14)
C15	0.0210 (18)	0.0250 (18)	0.0192 (17)	−0.0039 (15)	−0.0021 (15)	−0.0058 (15)
C16	0.0291 (18)	0.0173 (16)	0.0182 (16)	−0.0043 (13)	0.0024 (15)	−0.0043 (13)
C17	0.0249 (19)	0.0242 (19)	0.0216 (17)	0.0053 (15)	0.0030 (15)	−0.0054 (14)
C18	0.0186 (17)	0.0283 (19)	0.0239 (18)	0.0018 (15)	0.0061 (15)	−0.0032 (15)
C20	0.0289 (18)	0.0239 (17)	0.0175 (15)	−0.0004 (17)	−0.0029 (15)	0.0001 (14)
C21	0.0204 (17)	0.0257 (18)	0.0201 (16)	0.0025 (14)	−0.0065 (14)	0.0003 (14)
C22	0.0229 (18)	0.0272 (17)	0.0205 (16)	0.0019 (15)	−0.0026 (16)	0.0032 (13)
C23	0.0284 (19)	0.0210 (17)	0.0314 (19)	−0.0009 (14)	−0.0039 (16)	0.0031 (15)
C25	0.062 (3)	0.0220 (19)	0.036 (2)	−0.004 (2)	−0.002 (2)	−0.0032 (17)
C26	0.0268 (19)	0.0317 (19)	0.0204 (15)	0.0005 (17)	−0.0019 (14)	0.0030 (16)
As1A	0.0219 (2)	0.0175 (1)	0.0187 (1)	−0.0005 (1)	0.0006 (2)	−0.0038 (2)
O11A	0.0363 (15)	0.0273 (13)	0.0229 (12)	−0.0063 (12)	0.0047 (11)	−0.0041 (11)
O12A	0.0368 (14)	0.0204 (12)	0.0197 (11)	0.0008 (11)	0.0016 (10)	−0.0054 (10)
O13A	0.0239 (12)	0.0339 (14)	0.0505 (19)	0.0038 (11)	−0.0055 (13)	−0.0178 (13)
N4A	0.060 (3)	0.0279 (19)	0.070 (3)	0.0095 (18)	−0.011 (2)	−0.0174 (18)
C1A	0.0328 (19)	0.0171 (15)	0.0139 (15)	−0.0015 (15)	0.0001 (16)	−0.0007 (12)
C2A	0.0270 (19)	0.0250 (19)	0.031 (2)	0.0009 (15)	0.0045 (17)	−0.0009 (16)
C3A	0.042 (2)	0.026 (2)	0.028 (2)	0.0090 (17)	0.0073 (18)	0.0008 (16)
C4A	0.048 (3)	0.0208 (18)	0.0254 (18)	0.0027 (17)	−0.0018 (18)	0.0023 (15)
C5A	0.043 (2)	0.0239 (18)	0.0303 (19)	−0.0078 (19)	−0.0059 (19)	−0.0012 (15)
C6A	0.0313 (19)	0.0277 (18)	0.0224 (16)	−0.0030 (17)	−0.0018 (18)	−0.0022 (14)
O1W	0.0439 (18)	0.0500 (19)	0.0445 (18)	0.0115 (16)	0.0045 (15)	−0.0085 (16)
O2W	0.0453 (17)	0.053 (2)	0.058 (2)	0.0021 (17)	0.0002 (16)	0.0257 (18)
O3W	0.059 (2)	0.059 (2)	0.058 (2)	−0.0071 (19)	0.0088 (17)	−0.0055 (18)
O4W	0.050 (2)	0.0452 (19)	0.058 (2)	0.0023 (16)	0.0108 (17)	0.0184 (16)

### Geometric parameters (Å, º)

**Table:**

As1A—O12A	1.671 (2)	C13—C14	1.534 (4)
As1A—O13A	1.704 (2)	C14—C21	1.511 (5)
As1A—C1A	1.896 (3)	C14—C15	1.534 (5)
As1A—O11A	1.657 (2)	C15—C16	1.511 (5)
O2—C2	1.371 (4)	C17—C18	1.508 (5)
O2—C25	1.423 (4)	C20—C21	1.494 (5)
O3—C26	1.426 (4)	C21—C22	1.312 (5)
O3—C3	1.369 (4)	C22—C23	1.499 (5)
O24—C23	1.425 (4)	C1—H1	0.9500
O24—C12	1.423 (4)	C4—H4	0.9500
O25—C10	1.229 (4)	C8—H8	1.0000
O13A—H13A	0.90 (4)	C11—H111	0.9900
O1W—H12W	0.90 (3)	C11—H112	0.9900
O1W—H11W	0.90 (4)	C12—H12	1.0000
O2W—H22W	0.89 (3)	C13—H13	1.0000
O2W—H21W	0.90 (3)	C14—H14	1.0000
O3W—H32W	0.91 (3)	C15—H152	0.9900
O3W—H31W	0.91 (4)	C15—H151	0.9900
N9—C5	1.424 (4)	C16—H16	1.0000
N9—C10	1.351 (4)	C17—H171	0.9900
N9—C8	1.488 (4)	C17—H172	0.9900
N19—C16	1.529 (4)	C18—H182	0.9900
N19—C18	1.509 (4)	C18—H181	0.9900
N19—C20	1.503 (4)	C20—H202	0.9900
O4W—H42W	0.89 (4)	C20—H201	0.9900
O4W—H41W	0.90 (4)	C22—H22	0.9500
N19—H19	0.91 (4)	C23—H232	0.9900
N4A—C4A	1.374 (5)	C23—H231	0.9900
N4A—H41A	0.89 (3)	C25—H252	0.9800
N4A—H42A	0.90 (3)	C25—H251	0.9800
C1—C2	1.383 (5)	C25—H253	0.9800
C1—C6	1.393 (5)	C26—H262	0.9800
C2—C3	1.407 (5)	C26—H263	0.9800
C3—C4	1.381 (4)	C26—H261	0.9800
C4—C5	1.388 (4)	C1A—C6A	1.377 (5)
C5—C6	1.380 (4)	C1A—C2A	1.396 (5)
C6—C7	1.506 (5)	C2A—C3A	1.377 (5)
C7—C17	1.541 (4)	C3A—C4A	1.381 (6)
C7—C16	1.540 (5)	C4A—C5A	1.389 (6)
C7—C8	1.557 (5)	C5A—C6A	1.379 (5)
C8—C13	1.527 (5)	C2A—H2A	0.9500
C10—C11	1.511 (5)	C3A—H3A	0.9500
C11—C12	1.543 (5)	C5A—H5A	0.9500
C12—C13	1.537 (5)	C6A—H6A	0.9500
			
O12A—As1A—C1A	110.46 (12)	N9—C8—H8	110.00
O13A—As1A—C1A	101.45 (14)	C7—C8—H8	110.00
O12A—As1A—O13A	111.55 (13)	C13—C8—H8	110.00
O11A—As1A—C1A	112.41 (13)	C12—C11—H111	108.00
O11A—As1A—O12A	111.48 (11)	C10—C11—H111	108.00
O11A—As1A—O13A	109.09 (12)	C10—C11—H112	108.00
C2—O2—C25	117.1 (3)	H111—C11—H112	107.00
C3—O3—C26	116.2 (3)	C12—C11—H112	108.00
C12—O24—C23	114.5 (3)	O24—C12—H12	109.00
As1A—O13A—H13A	114 (3)	C13—C12—H12	109.00
H11W—O1W—H12W	102 (3)	C11—C12—H12	109.00
H21W—O2W—H22W	108 (5)	C8—C13—H13	107.00
H31W—O3W—H32W	100 (4)	C12—C13—H13	107.00
C8—N9—C10	120.1 (3)	C14—C13—H13	106.00
C5—N9—C10	125.0 (3)	C15—C14—H14	109.00
C5—N9—C8	109.1 (2)	C21—C14—H14	109.00
C16—N19—C18	107.3 (2)	C13—C14—H14	109.00
C16—N19—C20	112.9 (3)	H151—C15—H152	109.00
C18—N19—C20	112.3 (3)	C16—C15—H151	110.00
H41W—O4W—H42W	104 (4)	C16—C15—H152	110.00
C20—N19—H19	106 (2)	C14—C15—H151	110.00
C18—N19—H19	108 (3)	C14—C15—H152	110.00
C16—N19—H19	110 (3)	N19—C16—H16	108.00
H41A—N4A—H42A	130 (4)	C15—C16—H16	108.00
C4A—N4A—H42A	106 (3)	C7—C16—H16	109.00
C4A—N4A—H41A	119 (3)	C7—C17—H171	111.00
C2—C1—C6	119.1 (3)	C7—C17—H172	111.00
O2—C2—C1	124.6 (3)	C18—C17—H171	111.00
O2—C2—C3	115.5 (3)	C18—C17—H172	111.00
C1—C2—C3	120.0 (3)	H171—C17—H172	109.00
O3—C3—C2	115.5 (3)	H181—C18—H182	109.00
O3—C3—C4	123.3 (3)	C17—C18—H182	111.00
C2—C3—C4	121.2 (3)	N19—C18—H182	111.00
C3—C4—C5	117.7 (3)	C17—C18—H181	111.00
C4—C5—C6	122.1 (3)	N19—C18—H181	111.00
N9—C5—C4	127.7 (3)	C21—C20—H201	110.00
N9—C5—C6	110.1 (3)	N19—C20—H202	110.00
C5—C6—C7	110.5 (3)	H201—C20—H202	108.00
C1—C6—C7	129.4 (3)	N19—C20—H201	110.00
C1—C6—C5	119.9 (3)	C21—C20—H202	110.00
C6—C7—C8	102.5 (3)	C23—C22—H22	118.00
C16—C7—C17	102.0 (3)	C21—C22—H22	118.00
C6—C7—C17	112.4 (3)	O24—C23—H232	109.00
C8—C7—C17	110.8 (3)	O24—C23—H231	109.00
C6—C7—C16	115.4 (3)	H231—C23—H232	108.00
C8—C7—C16	114.1 (3)	C22—C23—H232	109.00
C7—C8—C13	116.6 (3)	C22—C23—H231	109.00
N9—C8—C7	104.5 (3)	H251—C25—H253	109.00
N9—C8—C13	106.0 (2)	H252—C25—H253	110.00
O25—C10—C11	120.7 (3)	H251—C25—H252	109.00
O25—C10—N9	122.5 (3)	O2—C25—H253	109.00
N9—C10—C11	116.8 (3)	O2—C25—H251	110.00
C10—C11—C12	118.1 (3)	O2—C25—H252	109.00
O24—C12—C11	105.3 (3)	O3—C26—H261	110.00
O24—C12—C13	114.4 (3)	H261—C26—H262	109.00
C11—C12—C13	109.9 (3)	H261—C26—H263	109.00
C8—C13—C12	106.8 (3)	H262—C26—H263	109.00
C8—C13—C14	112.0 (3)	O3—C26—H262	110.00
C12—C13—C14	117.8 (3)	O3—C26—H263	109.00
C13—C14—C15	106.0 (3)	As1A—C1A—C2A	119.6 (3)
C15—C14—C21	109.8 (3)	C2A—C1A—C6A	119.0 (3)
C13—C14—C21	114.4 (3)	As1A—C1A—C6A	121.4 (3)
C14—C15—C16	108.1 (3)	C1A—C2A—C3A	119.8 (4)
C7—C16—C15	115.7 (3)	C2A—C3A—C4A	121.7 (4)
N19—C16—C7	105.0 (3)	N4A—C4A—C5A	120.9 (4)
N19—C16—C15	110.5 (3)	N4A—C4A—C3A	121.2 (4)
C7—C17—C18	103.1 (3)	C3A—C4A—C5A	117.9 (4)
N19—C18—C17	105.1 (3)	C4A—C5A—C6A	121.1 (4)
N19—C20—C21	109.7 (2)	C1A—C6A—C5A	120.5 (4)
C14—C21—C20	114.6 (3)	C1A—C2A—H2A	120.00
C14—C21—C22	123.4 (3)	C3A—C2A—H2A	120.00
C20—C21—C22	122.0 (3)	C2A—C3A—H3A	119.00
C21—C22—C23	123.3 (3)	C4A—C3A—H3A	119.00
O24—C23—C22	111.9 (3)	C6A—C5A—H5A	119.00
C6—C1—H1	120.00	C4A—C5A—H5A	119.00
C2—C1—H1	120.00	C1A—C6A—H6A	120.00
C5—C4—H4	121.00	C5A—C6A—H6A	120.00
C3—C4—H4	121.00		
			
O11A—As1A—C1A—C2A	−51.9 (3)	C17—C7—C8—C13	−140.8 (3)
O11A—As1A—C1A—C6A	130.0 (2)	C6—C7—C16—N19	153.5 (3)
O12A—As1A—C1A—C2A	73.4 (3)	C8—C7—C16—N19	−88.2 (3)
O12A—As1A—C1A—C6A	−104.8 (3)	C8—C7—C16—C15	33.9 (4)
O13A—As1A—C1A—C2A	−168.3 (3)	C17—C7—C16—N19	31.3 (3)
O13A—As1A—C1A—C6A	13.6 (3)	C17—C7—C16—C15	153.4 (3)
C25—O2—C2—C1	1.0 (5)	C6—C7—C17—C18	−166.0 (3)
C25—O2—C2—C3	−178.9 (3)	C8—C7—C17—C18	80.1 (3)
C26—O3—C3—C2	178.7 (3)	C6—C7—C16—C15	−84.4 (4)
C26—O3—C3—C4	−1.1 (4)	C6—C7—C8—N9	−17.5 (3)
C23—O24—C12—C13	−69.2 (4)	C6—C7—C8—C13	99.1 (3)
C12—O24—C23—C22	87.0 (4)	C16—C7—C8—N9	−142.9 (3)
C23—O24—C12—C11	170.0 (3)	C16—C7—C8—C13	−26.3 (4)
C8—N9—C5—C6	−3.2 (4)	C17—C7—C8—N9	102.6 (3)
C8—N9—C5—C4	174.7 (3)	C16—C7—C17—C18	−41.8 (3)
C5—N9—C10—O25	−24.5 (5)	N9—C8—C13—C12	−71.7 (3)
C10—N9—C5—C4	22.1 (6)	N9—C8—C13—C14	158.0 (3)
C10—N9—C5—C6	−155.9 (3)	C7—C8—C13—C14	42.2 (4)
C5—N9—C8—C7	13.4 (3)	C7—C8—C13—C12	172.5 (3)
C5—N9—C8—C13	−110.4 (3)	O25—C10—C11—C12	150.9 (4)
C10—N9—C8—C7	167.6 (3)	N9—C10—C11—C12	−29.9 (5)
C10—N9—C8—C13	43.9 (4)	C10—C11—C12—C13	−0.2 (4)
C8—N9—C10—O25	−174.4 (3)	C10—C11—C12—O24	123.5 (3)
C8—N9—C10—C11	6.3 (5)	C11—C12—C13—C8	49.1 (3)
C5—N9—C10—C11	156.3 (3)	O24—C12—C13—C8	−69.1 (3)
C20—N19—C16—C15	−10.7 (4)	O24—C12—C13—C14	57.9 (4)
C16—N19—C18—C17	−16.7 (3)	C11—C12—C13—C14	176.1 (3)
C18—N19—C16—C7	−9.6 (3)	C12—C13—C14—C15	172.5 (3)
C18—N19—C16—C15	−134.9 (3)	C8—C13—C14—C15	−63.1 (3)
C20—N19—C16—C7	114.7 (3)	C8—C13—C14—C21	58.1 (4)
C18—N19—C20—C21	74.2 (3)	C12—C13—C14—C21	−66.4 (4)
C20—N19—C18—C17	−141.3 (3)	C15—C14—C21—C22	176.6 (3)
C16—N19—C20—C21	−47.3 (4)	C21—C14—C15—C16	−54.5 (3)
C2—C1—C6—C7	−174.0 (3)	C13—C14—C15—C16	69.6 (3)
C6—C1—C2—O2	−177.6 (3)	C15—C14—C21—C20	−4.2 (4)
C6—C1—C2—C3	2.3 (5)	C13—C14—C21—C20	−123.2 (3)
C2—C1—C6—C5	0.4 (5)	C13—C14—C21—C22	57.6 (5)
C1—C2—C3—C4	−3.1 (5)	C14—C15—C16—N19	62.7 (3)
O2—C2—C3—O3	−3.0 (4)	C14—C15—C16—C7	−56.4 (4)
O2—C2—C3—C4	176.9 (3)	C7—C17—C18—N19	36.4 (3)
C1—C2—C3—O3	177.1 (3)	N19—C20—C21—C14	56.0 (4)
O3—C3—C4—C5	−179.2 (3)	N19—C20—C21—C22	−124.8 (4)
C2—C3—C4—C5	1.0 (5)	C20—C21—C22—C23	177.7 (3)
C3—C4—C5—N9	−176.0 (3)	C14—C21—C22—C23	−3.2 (5)
C3—C4—C5—C6	1.8 (5)	C21—C22—C23—O24	−62.7 (5)
N9—C5—C6—C7	−9.0 (4)	As1A—C1A—C2A—C3A	−179.7 (3)
N9—C5—C6—C1	175.6 (3)	C6A—C1A—C2A—C3A	−1.6 (5)
C4—C5—C6—C1	−2.5 (6)	As1A—C1A—C6A—C5A	−179.2 (3)
C4—C5—C6—C7	172.9 (3)	C2A—C1A—C6A—C5A	2.7 (5)
C5—C6—C7—C16	141.2 (3)	C1A—C2A—C3A—C4A	−0.4 (5)
C1—C6—C7—C8	−168.6 (4)	C2A—C3A—C4A—N4A	179.6 (4)
C1—C6—C7—C16	−44.0 (5)	C2A—C3A—C4A—C5A	1.2 (5)
C1—C6—C7—C17	72.5 (5)	N4A—C4A—C5A—C6A	−178.5 (4)
C5—C6—C7—C8	16.6 (4)	C3A—C4A—C5A—C6A	−0.1 (5)
C5—C6—C7—C17	−102.4 (4)	C4A—C5A—C6A—C1A	−1.8 (5)

### Hydrogen-bond geometry (Å, º)

**Table:**

*D*—H···*A*	*D*—H	H···*A*	*D*···*A*	*D*—H···*A*
N19—H19···O12*A*	0.91 (4)	1.72 (4)	2.610 (3)	168 (4)
N4*A*—H41*A*···O4*W*^i^	0.89 (3)	2.46 (4)	3.291 (5)	155 (4)
N4*A*—H42*A*···O3*W*	0.90 (3)	2.25 (3)	3.137 (6)	169 (4)
O13*A*—H13*A*···O11*A*^ii^	0.90 (4)	1.67 (4)	2.546 (3)	165 (4)
O1*W*—H11*W*···O25	0.90 (4)	1.95 (4)	2.843 (4)	175 (3)
O1*W*—H12*W*···O2*W*^iii^	0.90 (3)	1.87 (4)	2.760 (5)	168 (4)
O2*W*—H21*W*···O12*A*	0.90 (3)	2.11 (3)	2.945 (4)	153 (4)
O2*W*—H22*W*···O11*A*^iv^	0.89 (3)	2.07 (4)	2.915 (4)	158 (5)
O3*W*—H31*W*···O25^v^	0.91 (4)	2.06 (4)	2.922 (4)	159 (3)
O3*W*—H32*W*···O4*W*^vi^	0.91 (3)	1.91 (3)	2.791 (4)	164 (3)
O4*W*—H41*W*···O1*W*^vii^	0.90 (4)	1.88 (4)	2.770 (5)	172 (5)
O4*W*—H42*W*···O12*A*	0.89 (4)	1.91 (4)	2.802 (4)	174 (5)
C4—H4···O25	0.95	2.37	2.900 (4)	115
C6*A*—H6*A*···O13*A*	0.95	2.55	3.011 (4)	110
C8—H8···O24	1.00	2.60	3.009 (4)	104
C14—H14···O3^viii^	1.00	2.52	3.363 (4)	142
C15—H151···O11*A*^ii^	0.99	2.60	3.561 (4)	165
C18—H182···O2*W*	0.99	2.58	3.422 (5)	143
C20—H201···O11*A*^ii^	0.99	2.41	3.388 (4)	170
C20—H202···O13*A*^iv^	0.99	2.43	3.229 (4)	137

Symmetry codes: (i) *x*−1/2, −*y*+3/2, −*z*+1; (ii) *x*+1/2, −*y*+1/2, −*z*+1; (iii) −*x*, *y*−1/2, −*z*+1/2; (iv) *x*−1/2, −*y*+1/2, −*z*+1; (v) −*x*+1/2, −*y*+1, *z*+1/2; (vi) *x*+1/2, −*y*+3/2, −*z*+1; (vii) −*x*+1, *y*+1/2, −*z*+1/2; (viii) −*x*+1, *y*−1/2, −*z*+1/2.
